# Application of conjugated materials in muscle movement recovery process

**DOI:** 10.3389/fchem.2023.1246926

**Published:** 2023-07-27

**Authors:** Dengfeng Zhang, Guanxi Fan

**Affiliations:** ^1^ Shandong Sport University, Jinan, Shandong, China; ^2^ School of Physical Education, Myongji University, Yongin-gu, Republic of Korea

**Keywords:** muscle movement recovery, conjugated materials, muscle contraction, application effect, photodynamic therapy

## Abstract

Conjugate materials have a good application effect in muscle movement recovery. This article aims to provide more references for the practical application of conjugated materials in sports recovery. This paper takes the students of the local physical education college as the experimental object, and selects the students who have sports muscle fatigue or injury for the test. In this paper, they are randomly divided into two groups: the experimental group and the control group, with 19 students in each group. The experimental group used the conjugate material in this paper for muscle movement recovery, while the control group used the traditional method for muscle movement recovery. This paper tested the peak torque, total work done, maximum radial displacement, and contraction time of two groups of students after initial exercise and muscle recovery. The experimental results showed that after 80 h of muscle movement recovery, the peak torque values of isometric contraction (264.59) and concentric contraction (160.81) of students in the experimental group were higher than those of students in the control group (233.79) and concentric contraction (130.43), and the difference was statistically significant (p < 0.05); the isometric contraction time (30.02) and concentric contraction time (29.31) of the experimental group were also higher than those of the control group (27.31) and concentric contraction time (24.58), which was statistically significant (p < 0.05). This study shows that conjugated materials have a significant effect on promoting muscle recovery. They not only help to increase the peak torque of muscle isometric contraction and concentric contraction, but also increase the time of muscle contraction and improve muscle mass.

## 1 Introduction

With the gradual penetration of the current concept of national fitness into people’s hearts, more and more people are paying attention to sports, which also makes the problem of muscle injury or muscle fatigue more prominent. High load, multifrequency, and high-intensity exercise can cause varying degrees of muscle damage or fatigue, which can affect training effectiveness, increase exercise risk, and decrease exercise level. Its main manifestation is a decrease in muscle strength, the inability of muscles to continue working, and a decrease in their ability to recruit muscle fibers. Intervention through intervention methods can to some extent accelerate the elimination of muscle injury and fatigue, and effectively restore the muscle motor function of athletes. Exercise muscle recovery can be intervened through different means. There are physical factor intervention, nutrition therapy, traditional Chinese medicine therapy, exercise therapy and drug therapy. Conjugated materials, as a material with excellent optical and self-assembly properties, have been widely used in the fields of imaging and diagnosis. Therefore, this article introduces conjugated materials into the process of muscle motion recovery, hoping that this method can better improve the effect of muscle motion recovery and ensure exercise safety. This can avoid the subsequent sports injury and fatigue, and provide more theoretical basis for the application of conjugated materials in the recovery of muscle movement.

As a huge and complex process, human movement is completed cooperatively under the domination and control of the central nervous system, in which muscles with contractive function are the power source of the motor system (Jiang et al., 2019). Therefore, muscle movement recovery has become a focus of attention for many scholars, who explore the methods and influencing factors of muscle movement recovery and propose different methods to promote muscle movement function recovery. Schambra, Heidi M linked the state of motor evoked potentials with coordination and recovery of movement between proximal and distal joints. Through testing, it was found that this method contributes to muscle contraction and strength recovery, especially in biceps training recovery (Schambra et al., 2019). Bayer, Monika L. aimed to investigate the effectiveness of tissue regeneration and early and delayed rehabilitation on the recovery of muscle motor function and structure after strain injury. Through experiments, it was found that muscle strain can damage tendon units, and structural and functional recovery is similar to early delayed rehabilitation, which is related to faster recovery of muscle motor function (Bayer et al., 2018). D’Amico Anthony P found through tests that after repeated sprints caused muscle sports injury, foam rolling seemed to speed up the recovery of muscle agility (D’Amico and Gillis., 2019). Tsuk, Sharon demonstrated through experimental testing that the inconsistent effects of photobiomodulation on muscle performance and muscle recovery after fatigue are due to differences among various variables, which can affect the results of photobiomodulation on muscle performance and exercise recovery (Tsuk et al., 2020). These scholars’ research on muscle recovery would help enrich its theoretical content and provide more possibilities for muscle restoration movement, but there are also some shortcomings. Although scholars’ research can promote muscle movement recovery to a certain extent, its recovery effect is not obvious, and the mechanism of promoting muscle movement recovery is also unclear, which leads to the inability of research to be applied well and has limitations.

Some scholars also turned their research direction to conjugated materials. They combined conjugated materials to study muscle mass, and said that Conjugated linoleic acid has a positive role in promoting muscle mass increase and enhancing sports endurance. Chang, Huan studied the effect of Conjugated linoleic acid on body composition of Chinese adults with increased body fat percentage, and found that Conjugated linoleic acid can increase trunk muscle mass compared with placebo (Chang et al., 2020). Duan, Chen said that Conjugated linoleic acid is related to the regulation of muscle fibers. He pointed out through experiments that Conjugated linoleic acid isomers increase the type of oxidized skeletal muscle fibers through toll like receptor 4 signals to enhance exercise endurance (Duan et al., 2021). Scholars’ research on conjugated materials and muscles can provide some theoretical support for this article, but due to the lack of emphasis on the application of conjugated materials in muscle motion recovery, the research does not have much reference value. From the research of these scholars, it can be seen that there is currently a lack of research on the application of conjugated materials in the process of muscle motion recovery, and more research is needed.

**FIGURE 1:**
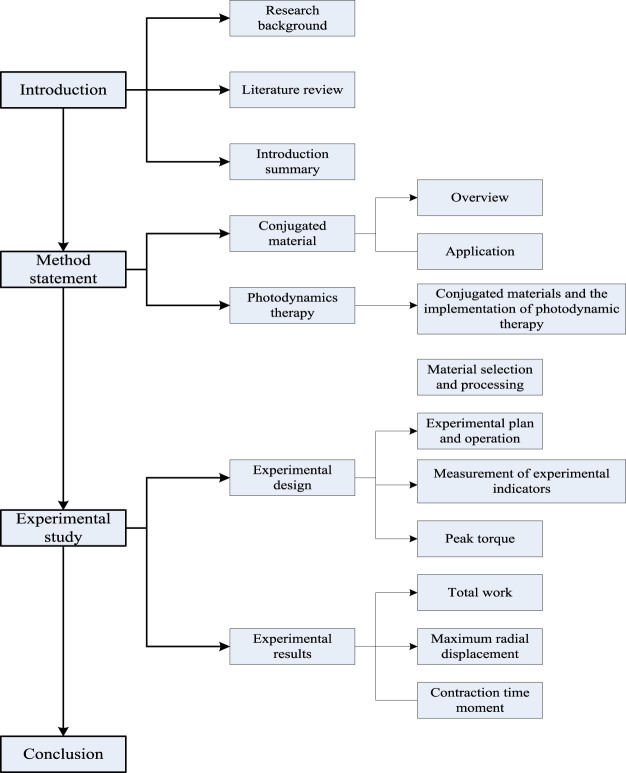
Research framework of this article.

In order to better promote muscle motion recovery, this article combines the theoretical analysis of conjugated materials and their applications with previous scholars’ discussions on muscle motion recovery, indicating that conjugated materials can be applied to the process of muscle motion recovery. Through empirical research, it is found that this method can effectively promote muscle motion recovery. Compared with traditional methods, the innovation of this method lies in its focus on the practical value of conjugated materials and their application in muscle recovery processes, which helps to achieve efficient muscle recovery and ensure exercise health. The framework of this study is shown in Figure 1:

## 2 Conjugated materials and applications

### 2.1 Conjugated materials

#### 2.1.1 Overview

Conjugated materials are a type of organism formed by the interweaving of benzene rings and double bonds in space (Zhao et al., 2019; Mateo-Alonso, 2023). In such compounds, electrons can freely migrate between the benzene ring and the double bond, forming a conjugated structure with π electrons (Song et al., 2019). This material has the characteristics of absorption and radiation, and has important applications in organic optoelectronic and electronic devices (Li et al., 2020). This article selects water-soluble conjugated materials as an example to analyze their application in the recovery of motor muscle function.

Water soluble conjugated materials are a typical amphiphilic structure due to their hydrophobic main chain and hydrophilic side chains (Zhou et al., 2019a; Bai et al., 2020). In aqueous solution, it can self assemble well to form supermolecule structure, micelles, nanoparticles, etc. Due to differences in external space size, solvents, temperature, and photoelectric stimuli, their self-assembly behavior can also be affected. Self assembly behavior leads to the formation of different aggregation states and conformations in conjugated materials, thereby exhibiting different optoelectronic properties. Through the thorough examination of molecular structure and external environment of conjugated materials, the foundation for the further development of conjugated materials is laid.

#### 2.1.2 Application

Water soluble conjugated materials have important applications in fields such as organic semiconductors, organic light-emitting diodes, and solar cells. Water soluble conjugated materials have many advantages, and the ionized functional groups in their side chains have good self-assembly performance, which is conducive to device preparation. They can significantly improve the electron injection efficiency from metal electrodes to organic luminescent layers. Due to its water-soluble properties, green and environmentally friendly solvents such as water and ethanol can be used for preparation. Water soluble organic conjugated materials have highly sensitive fluorescence effects and can achieve highly sensitive detection of various chemical or biological species (Sun and Schanze, 2022).

The fluorescence effect has made water-soluble conjugated materials widely used in fields such as fluorescent biosensors. In recent years, water-soluble conjugated items have been used extensively in fluorescence imaging of cells and *in vivo* through self-assembly with themselves or other small molecules. Since singlet state oxygen has a strong killing ability to bacteria, tumor cells, etc., it would use this new polymer material to stimulate oxygen, making it a new type of photodynamic therapy (Zhou et al., 2019b).

At present, in fields such as imaging and diagnosis, conjugated materials are often used as carriers, and their self-assembly properties are utilized to prepare multifunctional nanoparticles (Lu et al., 2018). In addition, the self-assembly behavior of self-assembly properties plays an important role in deeply understanding the optoelectronic properties, external environment, conformational changes, and other aspects of conjugated materials. The relationship between the self-assembly properties of cationic polyphenylene oxide and the changes in acidity and alkalinity indicates that conjugated materials exhibit different spectral properties in aqueous solutions with different acidity and alkalinity. Two completely different conformational ratios of conjugated materials can be clearly seen from the UV (ultraviolet) absorption spectra with different pH values. When the pH value changes from 6.2 to 7.0, the conformational migration of conjugated materials undergoes a significant change. From this, it can be seen that the reason why the acidity and alkalinity determine the spectral properties is the electrostatic repulsion of the ionic groups on the polymer branch chain, which leads to an increase in the polymer skeleton or a decrease in the twist angle. These effects alter the effective conjugation length of the polymer.

It can be seen that the application of water-soluble conjugated materials in muscle motion recovery has a certain theoretical basis. In the process of muscle motion recovery, conjugated materials can serve as photosensitizers to generate reactive oxygen species through light excitation. These reactive oxygen species can promote the metabolic activity of muscle cells, thereby accelerating the speed of muscle recovery.

### 2.2 Photodynamic therapy for muscle motion recovery based on conjugated materials

Photodynamic therapy is a non-invasive treatment method that mainly utilizes photosensitizers or reactive oxygen species induced by light irradiation (Jalali, 2022; Shi et al., 2023). Under certain band illumination conditions, conjugated materials can act as photosensitizers to undergo electron or energy transfer with oxygen in the environment, generating cytotoxic reactive oxygen species, such as hydrogen peroxide, hydroxyl radical, singlet state oxygen, etc. These short-lived reactive oxygen species can cause irreversible damage to important components (proteins, lipids, etc.) and nucleic acids in cells, thereby accelerating cellular metabolic activity. However, some studies have shown that intracellular photodynamic therapy can only act on a very small range, and can only cause minor damage to the cell structure in a very short distance. Therefore, it is necessary to effectively excite the photosensitizer within a wavelength range above 600 nm in order to have sufficient tissue penetration. Because the penetration depth of photodynamic therapy in near-infrared region is limited, it is not suitable for clinical depth treatment.

Assuming A is a Hilbert space (Hasan et al., 2019), it can be inferred from the norm derived from the inner product in A that A is also a Banach space (Ilkhan and Evren Kara, 2019). According to the definition of conjugate operators in Banach spaces, for any bounded linear operators R, $g\in A^{*}$ , and a∈A on A, there are:

$$\left(R^{*}g\right)\left(a\right)=g\left(Ra\right)$$ (1)

According to the p-theorem, there exists $b_{g}\in A$ , and for any a∈A , there exists $g\left(a\right)=\left(a,b_{g}\right)$ . On the other hand, for any a∈A , there exists $g\left(Ra\right)=\left(Ra,b_{g}\right)$ , because $R^{*}g\in A^{*}$ , there is $b_{R^{*}g}\in A$ , and for any a∈A , there is a formula:

$$R^{*}g\left(a\right)=\left(a,b_{R^{*}g}\right)$$ (2)

According to Formulas (1, 2), $\left(Ra,b_{g}\right)=\left(a,b_{R^{*}g}\right)$ , then:

$$\left(Ra,b_{g}\right)\neq \left(a,R^{*}b_{g}\right)$$ (3)

If for any a∈A , there is:

$$\left(Ra,b_{g}\right)=\left(a,R^{*}b_{g}\right)$$ (4)

Substituting γR for Formula (4) yields the formula:

$$\left(a,{\left(\gamma R\right)}^{*}b_{g}\right)=\left(\gamma Ra,b_{g}\right)=\gamma \left(Ra,b_{g}\right)=\gamma \left(a,R^{*}b_{g}\right)=\left(a,{\bar{\gamma }R}^{*}b_{g}\right)$$ (5)

Among them, γ is a non zero complex number.

Furthermore, it can be concluded that:

$$\left(a,{\left(\gamma R\right)}^{*}b_{g}-{\bar{\gamma }R}^{*}b_{g}\right)=0$$ (6)

It can be seen from the arbitrariness of a:

$${\left(\gamma R\right)}^{*}b_{g}={\bar{\gamma }R}^{*}b_{g}$$ (7)

Due to the conjugated linear isomorphism meaning, A and $A^{*}$ are equal, which helps to promote the optical properties of conjugated materials to be better utilized in photodynamic therapy, thereby improving the effect of conjugated materials on muscle motion recovery.

## 3 Empirical study on conjugated materials in the recovery of muscle movement

### 3.1 Experimental design

Before conducting experimental analysis on the effects of conjugated materials on muscles, it is necessary to first select suitable conjugated materials as experimental research objects, process them accordingly, design and operate experiments, and finally analyze the experimental results.

#### 3.1.1 Material selection

Water soluble conjugated materials have unique photoelectric properties and water solubility that is friendly to biological tissues. On this basis, this paper takes rigid tetraphenylmethane and P-Xylene dimer as the core, fluorene, double bond, benzothiazole group as the branch chain. It can utilize its excellent photoelectric properties and good biological tissue affinity to achieve the repair of muscle motor function. On the one hand, narrow bandgap benzothiadiazole molecules have excellent carrier transport properties due to their planar rigid structure. It can also form a conjugated structure with the electron group donor acceptor, reduce its energy band gap, and broaden its spectral absorption range. Double bonds are a planar type of bond that is introduced into the main chain of molecules, making the main chain structure more planar, thus facilitating molecular absorption and emission. The two-photon structure of D-π-A-π-D formed with fluorene, benzothiadiazole, double bond, etc., as the center, has constructed a new type of structural unit that may produce Two-photon absorption. Later, this paper uses multi branched water-soluble conjugated materials with three-dimensional dendritic structure to maintain their relatively stable molecular morphology under the internal and intermolecular force, thus inhibiting their aggregation and quenching and ensuring their fluorescence quantum. If the material has Two-photon absorption property at the same time, its Two-photon absorption effect can be further enhanced. Rigid cores such as Tetraphenylmethane and P-Xylene dimer can be introduced into molecular design to reduce the aggregation, folding or twisting degree of the main chain through their volume effect. This can ensure the purity and quality of conjugated materials, maintain and improve their quantum efficiency and photon absorption effect.

#### 3.1.2 Material handling

**FIGURE 2:**
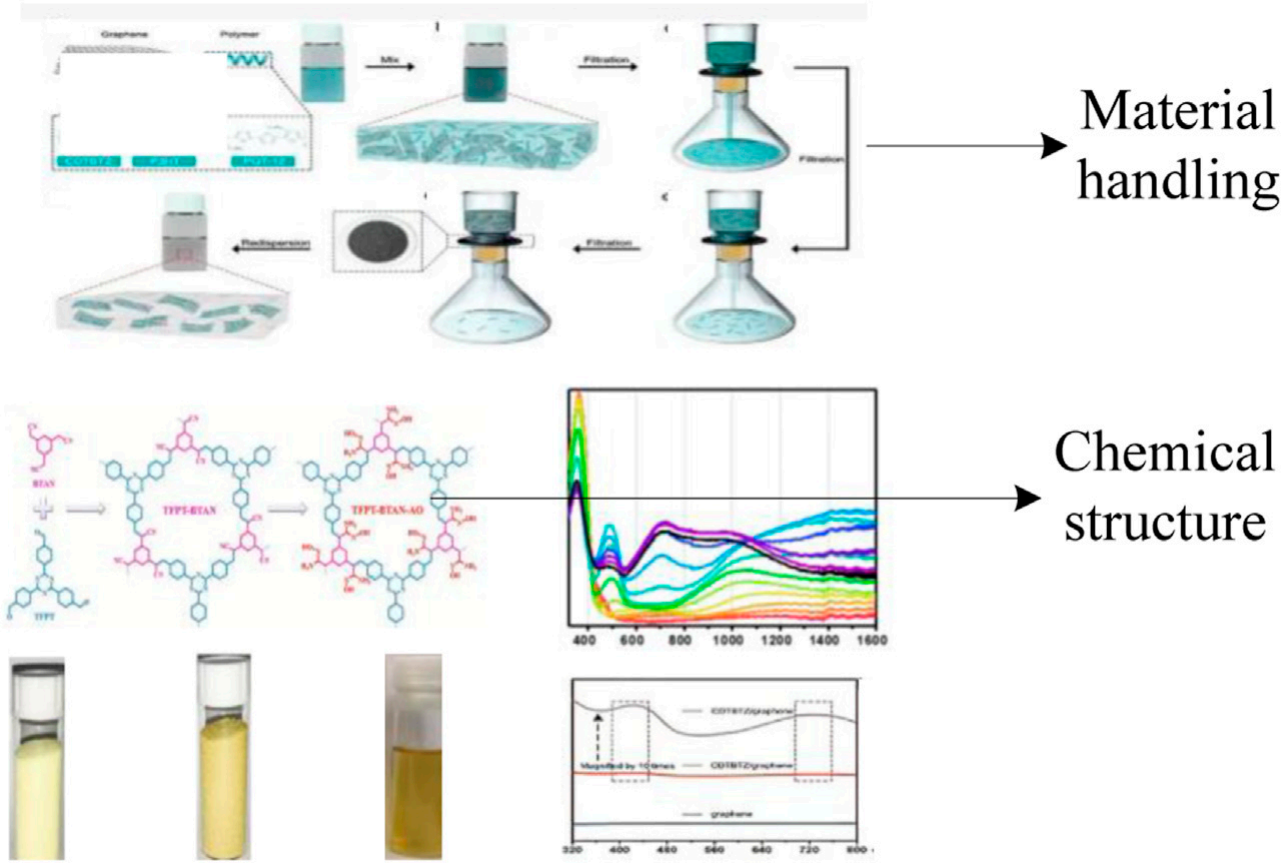
Material after treatment.

By reducing the production cost and the polymerization of dihydroxyacetone phosphate, it can easily and quickly obtain the near infrared water-soluble conjugated materials with good photothermal/photodynamic synergy. These water-soluble conjugated materials are substituted with alkylated pyrrole diketone derivatives at the 2,5 position, grafted with polyethylene glycol chains, dithiophene and fluorene derivatives, and grafted with macromolecular polyethylene glycol chains as polymerization monomers. By adjusting the monomer ratio, water-soluble conjugated materials are synthesized. The processed material is shown in Figure 2:

#### 3.1.3 Research methods

In order to better confirm the practical effect of using conjugated materials in the process of muscle exercise recovery in this article, 38 students from a local college of physical education were selected as the experimental subjects and tested for muscle fatigue or injury during exercise. This article randomly divides them into two groups: the research group and the control group, with 19 students in each group. The research group used the conjugated materials in this article for muscle motion recovery, while the control group used traditional methods for muscle motion recovery. There was no significant difference between the two groups of study subjects. The ages of the two groups of research subjects are between 19 and 22 years old; healthy body, no exercise taboos; the study subjects did not smoke, drink alcohol, consume caffeine, or engage in vigorous exercise within 24 h, and there was no significant difference between the two groups of study subjects (*p* > 0.05). The subjects willingly agreed to participate in the study after reading and signing an informed consent form and having a thorough grasp of the study’s objectives and procedures. The basic information of the two groups of students is shown in Table 1:

**TABLE 1:** Comparison of basic information between two groups of students.

Serial number	General situation	Study group		Control group	
1	Gender	Male	Female	Male	Female
2		13	6	14	5
3	Average age	20.13±2.11		20.56±1.87	
4	Body weight (kilogram)	65.19±7.88		66.42±6.29	
5	Height (meters)	1.71±0.34		1.75±0.15	
6	Body fat (%)	18.86±4.23		17.56±6.15	
7	Body mass index (kilograms per square meter)	25.16±1.71		24.21±1.02	
8	Maximum squat load (kilogram)	62.19±4.31		63.06±3.09	

#### 3.1.4 Experimental plan

This article measures the basic information of the subjects 7 days before the formal trial begins. The basic information for measurement includes height, weight, percentage of body fat, and maximum squat weight. This article uses TMG (Tensiomyography) technology to measure the peak torque value, total work, maximum radial displacement, and contraction time of the lower limb muscles of the experimental subjects, and uses the measurement results as the initial values before the experiment.

Two groups of students underwent 3-min stretching exercises at different time intervals after muscle recovery. This article measures the muscle function recovery of students at 1 h, 10 h, 20 h, 30 h, 40 h, 50 h, 60 h, 70 h, and 80 h after recovery.

#### 3.1.5 Indicator measurement

The peak torque (PT), total work (TW), maximum radial displacement (Displacement), and contraction time of each group of muscles were measured using TMG technology. TMG technology is a non traumatic test method, which is used to study the characteristics of Muscle contraction. It is mainly used to analyze the type of muscle fibers in a certain surface muscle, as well as the damage, fatigue, or tension state of the tested muscle. It can evaluate muscle function and also query the symmetry of morphology. In clinic, TMG technology can also quickly diagnose muscle sports injury within a period of time when it occurs, and continuously monitor the follow-up rehabilitation.

#### 3.1.6 Experimental operations

The extraction of compounds is monitored using a layer chromatography silica gel plate for point plate monitoring, and then purified through a chromatographic column. Based on the polarity of the product, a self-developed agent is prepared. Synthetic compound: the toluene solution formed by 2-bromofluorene and tetrabutyl ammonium bromide can be added dropwise to the potassium hydroxide aqueous solution, and stirred at room temperature for more than 20 min. Afterwards, it adds tert butyl acrylate dropwise and can be stirred at room temperature for 5 h. The mixture can then be diluted with 15 mL of dichloromethane and washed with water. Next, the organic layer is dried by anhydrous sodium sulfate. The solvent can be extracted by rotary evaporation method and the purified product can be obtained through a chromatographic column. It uses a mixed solvent as the eluent to obtain a light yellow solid product. 0.9% sodium chloride solution can be added into the precipitated cells for fluorescence detection under the laser confocal microscopy. Fluorescent labeled cells can be suspended in a 0.9% sodium chloride solution and cell density can be adjusted.

#### 3.1.7 Result statistics

The data analysis was conducted using SPSS 19.0. In this paper, the mean ± standard deviation (±s) is used to express the data that fit to the normal distribution, and the *p*-value (a parameter used to judge the hypothesis test results) is used between groups for grouping comparison. *p* < 0.05 indicates that the difference is statistically significant.

### 3.2 Result analysis

#### 3.2.1 Peak torque variation results

**FIGURE 3:**
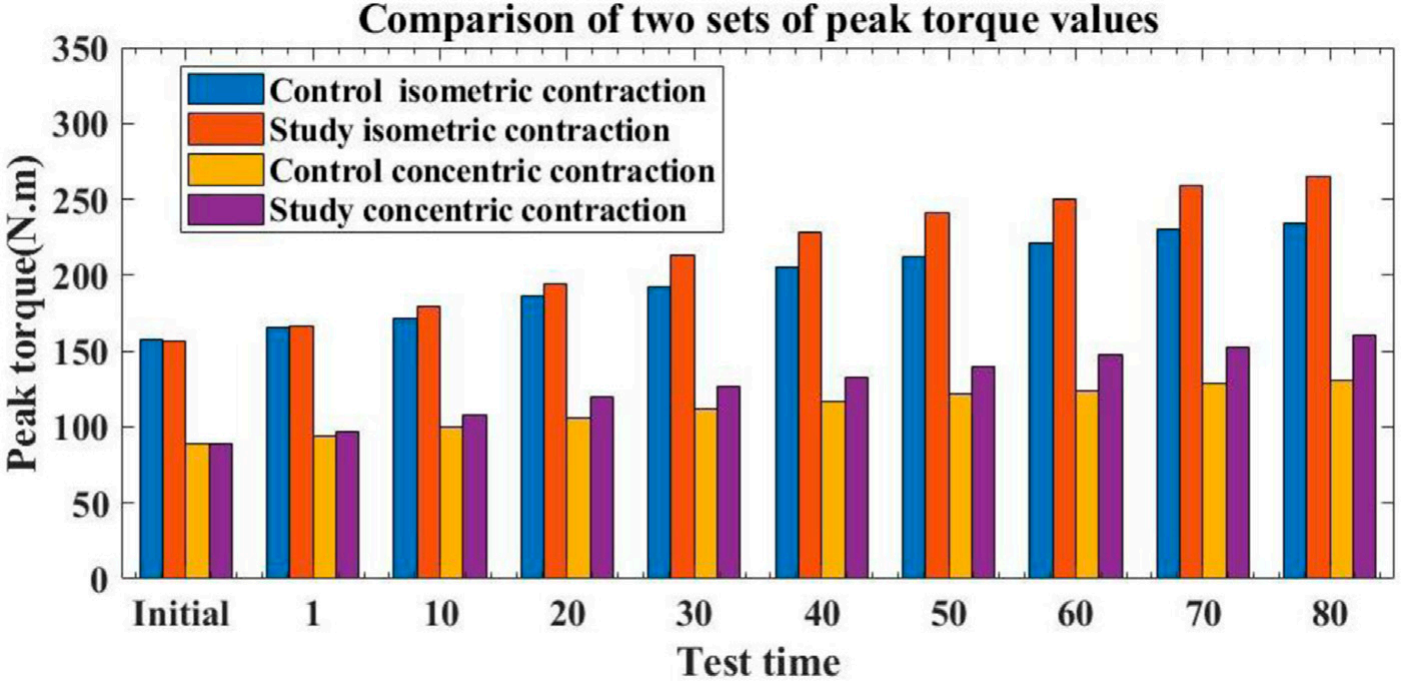
Peak torque changes of two groups of muscle sports injury and fatigue students at the initial stage of the experiment and at different periods after the experiment.

Peak torque, also known as maximum torque, reflects the absolute strength of the muscle and can effectively evaluate its maximum strength. This paper compares the peak torque changes of muscle sports injury and fatigue students during the same length contraction and centripetal contraction at the initial stage of the experiment and at different time periods after the experiment. The results are shown in Figure 3: (The first abscissa is the initial test time; 1 to 80 is the test time after the recovery of sports muscles; the ordinate is the peak torque value):

From Figure 3, it can be seen that the peak torque value of the initial isometric contraction of the control group students was 157.32, while the peak torque value of the initial isometric contraction of the experimental group students was 156.26. In the initial peak torque value of centripetal contraction, the control group was 89.31; the research group was 89.02. It can be seen that in the initial stage, no statistically significant difference was present in the peak torque values of isometric contraction and centripetal contraction (*p* > 0.05), indicating comparability of the study. After 1 h of muscle movement recovery using traditional materials, the control group students had a peak torque value of 165.63 for isometric contraction and 94.22 for centripetal contraction. The experimental group students had a peak torque value of 166.37 for isometric contraction after 1 h of muscle movement recovery using conjugated materials in this article, while the control group students had a peak torque value of 166.37. The peak torque value of centripetal contraction was 96.52. There was no significant increase in the peak torque of isometric contraction and centripetal contraction between the two groups of students, and the difference was not statistically significant (*p* > 0.05). This indicates that after 1 hour of treatment with different methods, the muscle movement recovery effect of the two groups of students was not significant and there was no significant improvement. After 80 h of muscle movement recovery using traditional materials, the peak torque values of isometric contraction for the control group students were 233.79 and 130.43, respectively. After 80 h of muscle movement recovery using conjugated materials in this article, the peak torque of isometric contraction was 264.59, and the peak torque of centripetal contraction was 160.81. Compared to traditional methods, the method proposed in this article showed a 30.8% increase in the peak torque of isometric contraction in the experimental group compared to the control group 80 h after exercise recovery; the peak torque value of centripetal contraction was 30.38 higher than that of the control group. There was a huge difference in peak torque, with statistical significance (*p* < 0.05). This shows that the method in this paper is more conducive to the recovery of absolute muscle strength of Student activism after sports muscle injury.

#### 3.2.2 Total power

**FIGURE 4:**
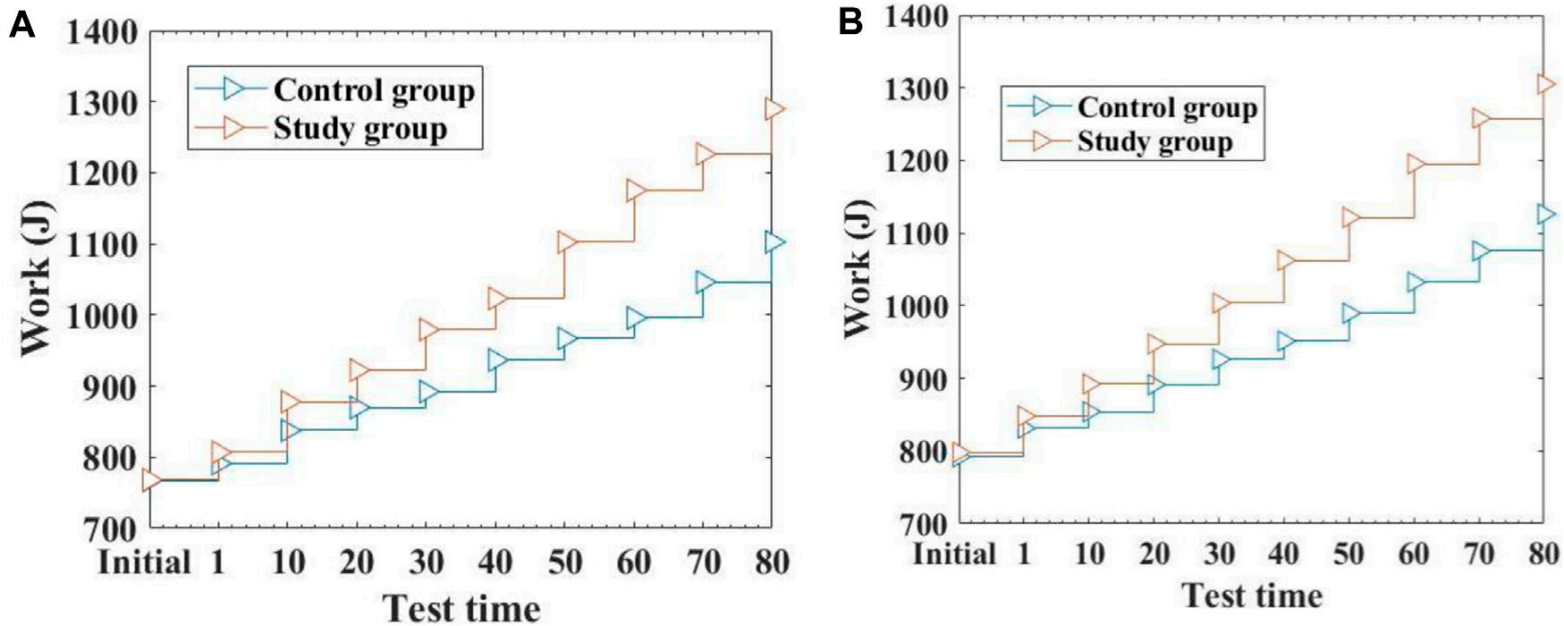
Comparison of the Results of Two Groups of Students Doing Work Different Times **(A)**: Comparison of the changes in work done by two groups of students during one concentric contraction **(B)**: Comparison of the changes in work done during five concentric contractions between two groups of students.

Total work reflects the total energy output and work ability of muscles, which is a comprehensive evaluation of the overall strength of muscles. This article compares the total work changes of one and five concentric contractions between two groups of students. The specific results are shown in Figure 4: (The horizontal axis represents the test time, and the vertical axis represents the work done):

From Figure 4A, it can be seen that the average value of initial centripetal contraction work done by the control group students was 767.34, while the experimental group was 768.21. There is no significant difference in the initial centripetal contraction work between the two groups of students, and it is comparable. After 80 h of muscle exercise recovery using traditional methods, the average work done by a single centripetal contraction in the control group was 1,102.55, while the average work done by a single centripetal contraction in the experimental group was 1,289.74 after 80 h of muscle exercise recovery using this method. Compared to traditional methods, the average work done by the experimental group students in one concentric contraction after muscle exercise recovery was 187.19 higher than that of the control group. The experimental group did significantly more work on one concentric contraction than the control group, with a statistically significant difference (*p* < 0.05). From Figure 4B, it can be seen that there is no significant difference in the initial five concentric contractions of the two groups of students. After 80 h of muscle exercise recovery through traditional methods, the average value of five concentric contractions performed by the control group reached 1,126.13. The average work done by the research group during five concentric contractions reached 1,305.21. Compared with the control group, the average value of five concentric contractions in the experimental group was 179.08 higher, and there was a huge difference in the average value of five concentric contractions between the two groups, which was statistically significant (*p* < 0.05). From it, it can be seen that the research group under the method of this article performed better in terms of overall muscle strength. This is because this article promotes the recovery of student muscle movement through conjugated materials and improves the recovery effect of student muscle movement.

#### 3.2.3 Comparison of maximum radial displacement of muscle movements

**FIGURE 5:**
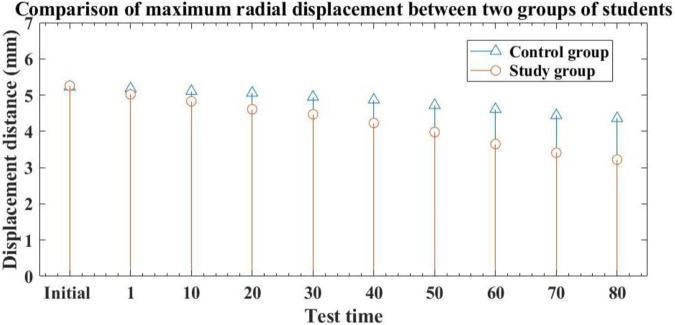
Comparison of the maximum radial displacement results of muscle movements between two groups of students at the beginning of the experiment and after recovery.

The maximum radial displacement of a muscle refers to the maximum amount of lateral displacement of the muscle during impact motion, which also indicates the hardness of the muscle, and even the degree of muscle tension. Compared with the database average, the relatively low maximum radial displacement value indicates that the measured muscles are more developed or have a better degree of stiffness. A higher value indicates that the measured muscle development is poor, or there is muscle fatigue or injury. It is an important standard for measuring the recovery effect of muscle movement. The test results of the maximum radial displacement of the left knee extensor muscle group during the initial and post recovery experiments for two groups of students are shown in Figure 5: (The horizontal axis represents the test time, and the vertical axis represents the maximum radial displacement distance):

From Figure 5, it can be seen that the maximum radial displacement of the control group students’ muscle movement at the initial time was 5.23 mm, while the research group was 5.26 mm. There is no significant difference in the maximum radial displacement of muscle movement between the two groups of students, indicating comparability. After traditional methods of exercise recovery, the maximum radial displacement of the control group students was 4.87 after 40 h of exercise recovery, and 4.36 after 80 h of exercise recovery. It can be seen that after using traditional methods for muscle movement recovery, the maximum radial displacement distance of the control group students’ muscle movement shortened relatively slowly, indicating that the traditional method has limited effect on muscle fatigue and injury recovery, and its effect is not significant. After performing exercise recovery using the method described in this article, the maximum radial displacement of the experimental group students after 40 h of exercise recovery was 4.23, and the maximum radial displacement after 80 h of exercise recovery was 3.22. Overall, the experimental group and the control group showed a significant reduction in the maximum radial displacement distance of muscle movement, indicating that the method proposed in this paper has a significant effect on alleviating muscle fatigue and promoting muscle injury recovery. Compared with the control group after 80 h of exercise recovery, the maximum radial displacement of the experimental group students decreased by 1.14. The maximum radial displacement of the experimental group after 80 h was significantly lower than that of the control group, with a statistically significant difference (*p* < 0.05).

#### 3.2.4 Comparison of muscle contraction time

**FIGURE 6:**
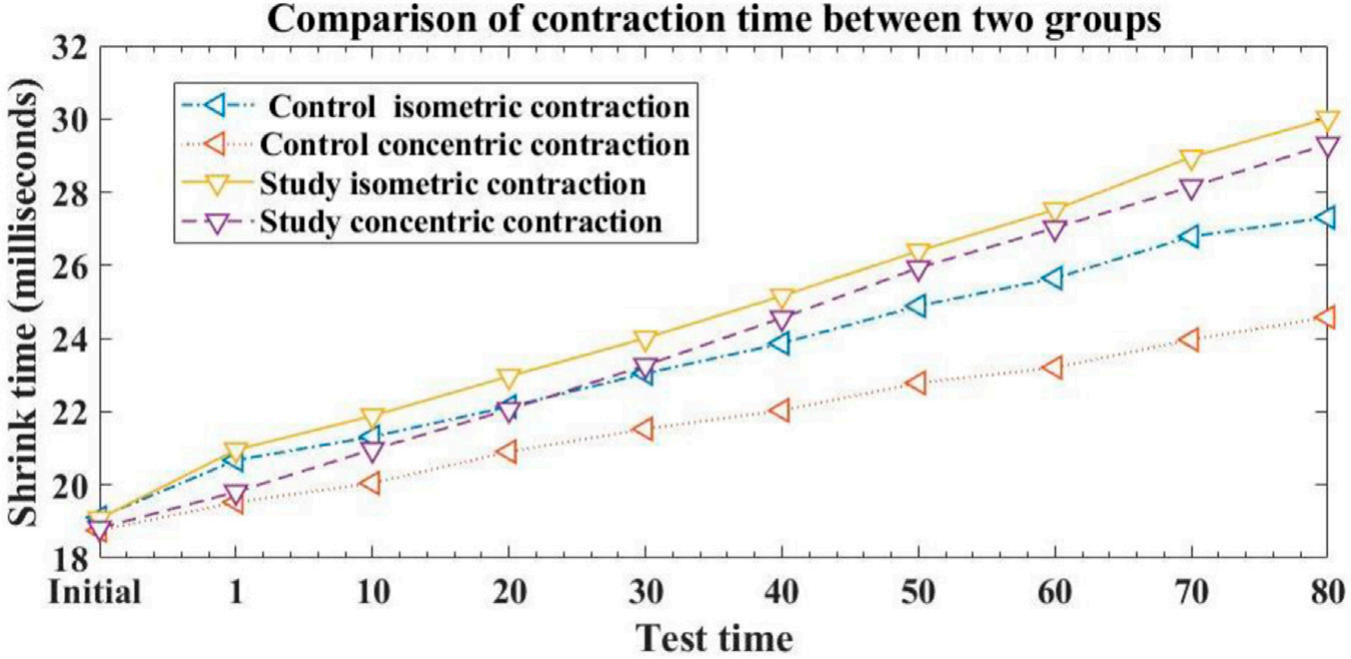
Comparison of muscle contraction time between two groups of students in different time periods.

In addition to maximum radial displacement, contraction time is also an indicator of muscle recovery. Generally speaking, the longer the contraction time of muscles, the better their quality, and the easier it is to avoid muscle damage. This article compared the isometric and centripetal muscle contractions of two groups of students who underwent stretching exercises at different time periods. The results are shown in Figure 6:

From Figure 6, it can be seen that the initial isometric contraction time of the control group was 19.11 milliseconds, and the centripetal contraction time was 18.76 milliseconds. The initial isometric contraction time of the research group was 19.08 milliseconds, and the centripetal contraction time was 18.82 milliseconds. There was no difference in the initial isometric contraction and centripetal contraction time between the two groups of students, and there was no statistical significance (*p* > 0.05), indicating comparability. 80 h after muscle movement recovery, the control group students in the traditional method had an isometric contraction time of 27.31 milliseconds and an centripetal contraction time of 24.58 milliseconds. The experimental group students under this method had an isometric contraction time of 30.02 milliseconds and an centripetal contraction time of 29.31 milliseconds. Compared to traditional methods, the experimental group of students who used this method for muscle movement recovery increased their isometric muscle contraction time by 2.71 milliseconds and their centripetal contraction time by 4.73 milliseconds at 80 h. The duration of isometric and centripetal contractions in the experimental group was significantly higher than that in the control group, with a statistically significant difference (*p* < 0.05). This means that using the method proposed in this article for muscle exercise recovery can help promote an increase in muscle protein synthesis, thereby improving the effectiveness of muscle exercise recovery.

### 3.3 Discussion

Muscle pain caused by sports injury is one of the main factors affecting human health. Many studies on the mechanism of muscle injury have shown that during intense exercise and severe injury, a large amount of free radicals are produced. During this process, the pore size and permeability of the cell membrane increase, leading to the destruction of capillary endothelial cells, causing inflammatory reactions and degenerative changes, and ultimately leading to skeletal muscle damage. Water soluble conjugated materials can act as photosensitizers, forming reactive oxygen species under light conditions. This reactive oxygen species can accelerate the metabolism of muscle cells and accelerate muscle recovery. Water soluble conjugated materials refer to main chains with π electron conjugated structures and molecules with negative ions, cations, or other hydrophilic groups. Structurally, water-soluble conjugated materials typically consist of two components, one of which is the π conjugated backbone, and their electron transport characteristics determine their optoelectronic properties. The other type is a charged or water-soluble side chain, such as quaternary ammonium cation group, carboxyl group, sulfonic acid group, phosphate group and polyethylene glycol chain. These groups enhance the solubility of substances and facilitate their functions in organisms, microorganisms, and cells. Due to the large π bond of conjugated materials, they have high light absorption ability in the UV visible region and high electron and energy transfer rates. On the other hand, by connecting ionic groups or other hydrophilic groups to the side chains of the Conjugated system, water solubility can be achieved, thus reacting with ionic biomolecules through electrostatic interaction.

In the experiment, the peak torque values of isometric contraction time and centripetal contraction time in the research group based on water-soluble conjugated material muscle motion recovery after 80 h were better than those in the control one based on traditional methods. At the same time, the maximum radial displacement distance of muscle movement in the research group after 80 h was significantly better than that in the control one. This indicates that conjugated materials can reduce metabolites and inflammatory substances in muscle tissue during muscle recovery, clearing obstacles during injury or fatigue recovery. It can increase the oxygen and blood supply in muscles, increase muscle enzyme activity, and create conditions for muscle energy synthesis. It can also adjust the microenvironment around muscle tissue, making it have good self-healing function. It can also stretch muscles, adjust their functional length, and facilitate muscle self recovery.

## 4 Conclusion

With the development of spectroscopy of conjugated materials, there would be more and more researches on the application of conjugated materials in motion recovery. The theme of this article is the application of conjugated materials in muscle motion recovery. Firstly, the background and significance of muscle motion recovery are introduced. Then this article summarized the research of previous scholars, elaborated on conjugated materials and their applications, and introduced conjugated materials into the process of muscle motion recovery. Finally, to verify its practical application effect, this article found through comparative research that the peak torque values of muscle isometric contraction time, centripetal contraction time, and maximum radial displacement distance of muscle movement in the research group using conjugated materials were superior to traditional methods. Its muscle movement recovery effect is better, which is more conducive to muscle recovery after exercise, and also has a certain role in preventing subsequent muscle damage and muscle fatigue. The selection of research subjects in this article is relatively small, and the number of reference samples is relatively small, which may have a certain impact on the final research results. In addition, there is less research on the aggregation fluorescence quenching, visible absorption and emission, and targeted localization of conjugated materials in this article. In future research, more in-depth exploration of these aspects is emphasized.

## Data Availability

The original contributions presented in the study are included in the article/supplementary material, further inquiries can be directed to the corresponding author.
